# Millipede Taxonomy after 250 Years: Classification and Taxonomic Practices in a Mega-Diverse yet Understudied Arthropod Group

**DOI:** 10.1371/journal.pone.0037240

**Published:** 2012-05-15

**Authors:** Michael S. Brewer, Petra Sierwald, Jason E. Bond

**Affiliations:** 1 Department of Biology, East Carolina University, Greenville, North Carolina, United States of America; 2 Department of Zoology, The Field Museum, Chicago, Illinois, United States of America; 3 Department of Biological Sciences, Auburn University Museum of Natural History, Auburn University, Auburn, Alabama, United States of America; Barnard College, Columbia University, United States of America

## Abstract

**Background:**

The arthropod class Diplopoda is a mega-diverse group comprising >12,000 described millipede species. The history of taxonomic research within the group is tumultuous and, consequently, has yielded a questionable higher-level classification. Few higher-taxa are defined using synapomorphies, and the practice of single taxon descriptions lacking a revisionary framework has produced many monotypic taxa. Additionally, taxonomic and geographic biases render global species diversity estimations unreliable. We test whether the ordinal taxa of the Diplopoda are consistent with regards to underlying taxonomic diversity, attempt to provide estimates for global species diversity, and examine millipede taxonomic effort at a global geographic scale.

**Methodology/Principal Findings:**

A taxonomic distinctness metric was employed to assess uniformity of millipede ordinal taxa. We found that ordinal-level taxa are not uniform and are likely overinflated with higher-taxa when compared to related groups. Several methods of estimating global species richness were employed (Bayesian, variation in taxonomic productivity, extrapolation from nearly fully described taxa). Two of the three methods provided estimates ranging from 13,413–16,760 species. Variations in geographic diversity show biases to North America and Europe and a paucity of works on tropical taxa.

**Conclusions/Significance:**

Before taxa can be used in an extensible way, they must be definable with respect to the diversity they contain and the diagnostic characters used to delineate them. The higher classification for millipedes is shown to be problematic from a number of perspectives. Namely, the ordinal taxa are not uniform in their underlying diversity, and millipedes appear to have a disproportionate number of higher-taxa. Species diversity estimates are unreliable due to inconsistent taxonomic effort at temporal, geographic, and phylogenetic scales. Lack of knowledge concerning many millipede groups compounds these issues. Diplopods are likely not unique in this regard as these issues may persist in many other diverse yet poorly studied groups.

## Introduction

The Linnean system of biological classification is an information-rich organizational scheme that has been in existence for over 250 years. Despite its antiquity, it remains, even today, the framework on which almost every biological question relies; it is the principal mode for communicating species level data within a relational and hierarchical context. Contained within the Linnean hierarchical schema is a wealth of information from which we can draw inferences regarding the tempo, mode, and pattern of evolutionary diversification. These classifications are fundamentally a hypothesis of phylogeny and can be used in conjunction with fossil and biogeographic data to make hypotheses concerning evolutionary processes that can be tested using modern phylogenetic techniques. In short, our present day system of biological classification, and the taxonomy on which it is based, infers much more than just categorical information for the purposes of simply “cataloging” taxa. Classification schemes, derived from modern systematic research, are fundamentally a hypothesis of phylogeny and thus provide a foundation for any ecological and/or evolutionary study.

Although a phylogenetic-based classification provides an evolutionary scaffolding, it is important to recognize that the hierarchical component of this framework is a product of its history and is often an intrinsic property of the study group. Therefore, classifications supported by phylogenies may or may not be internally consistent for any given taxonomic group contained therein and are likely inconsistent when compared to other organismal groups. That is, higher order taxonomic ranks (genera, families, orders, classes, phyla) are not necessarily equivalent (in regards to phylogenetic diversity, species diversity, morphological diversity, age, etc.) among animal groups. Inequality between taxonomic groups may stem from a number of factors including disparity in species diversity, phylogenetic relatedness, age, and degree of phenotypic divergence. For example, Warwick and Somerfield [1], using a taxonomic distinctness metric [2], showed that the marine phyla off the coast of Great Britain did not share similar amounts of diversity and thus lacked equivalency. For species found off the coast of Great Britain, a metric representing the average distance between species in a phylum, in a taxonomic context, was plotted against the number of species representing each phylum. A linear regression showed that these higher-level taxa were not equally diverse for the ecosystem.

While the fields of ecology and evolution rely heavily on accurate phylogenetic and classification frameworks, conservation related decisions require a standard metric to evaluate areas of priority. Species richness is the primary “yardstick” used to assess biodiversity. At some level, this assumes that species richness, as opposed to phylodiversity, ecosystem diversity, or genetic diversity, is the most meaningful indicator of diversity. Alternatively, a number of methods have been employed to estimate species diversity within a poorly studied geographical region using the number of higher taxa surveyed from the area and extrapolating based on some assumptions regarding the ratio of species to higher taxa [3], [4]. At the core of this argument is the assumption that higher taxa are equally diverse which is often not the case within or between taxonomic groups. Recent studies have applied this method to spiders (Arachnida: Araneae) and marine invertebrates with the best performance coming from the use of genera [5], [6]; however, these techniques can fail to make useful predictions [7]. Reliable estimates can only be made in taxa that are well described, in terms of percentage of nominal species, and/or have relatively small geographic areas. Other proposed approaches that rely on higher taxa include cladistic methods [8] and methods that use unique characteristics of species [9], which vary more across higher taxa, as the currency of biodiversity.

Regardless of disparities among the higher-level taxonomic categories between taxa, these groupings may provide some insight into the amount of biodiversity composing an ecological community. Given that species distributed among higher taxa potentially differ more in, for example, morphological, physiological, behavioral, and genetic characteristics than those that are closely related, the protection of higher taxa should be one of the factors of consideration for conservation planning and biodiversity assessment. While these higher-level groups may be defined on the basis of a set of uniquely derived characters (synapomorphies in cladistics parlance), placement of a rank on a phylogeny is thought to be facultative in regards to identification or ease of use. Actions such as these have led to the opinion that genera, families, orders, etc., are nebulous and likely represent artificial constructs [4], [10] (i.e., facultative placeholders). Although one might argue that higher taxa are simply epistemeological constructs, higher taxa defined on the basis of some metric, such as underlying diversity, may enhance their utility for assessments of biodiversity and conservation planning.

### Study group

The Diplopoda are a mega-diverse group of arthropods that are among the most important consumers of detritus in many terrestrial ecosystems. Comprising more than 12,000 described species (Table 1: though Shear [11] counted 7,753 valid species), millipedes are found on six continents and in virtually all of Earth's biomes [12]. Despite being conspicuous and abundant in most habitats, the group is understudied in many regards. Relative to their diversity in temperate and tropical ecosystems, there are few studies documenting aspects of the group's phylogeny, evolution, behavior, physiology and ecology. And, taxonomically, the group has an inconsistent and tumultuous history. As many as 300 species and as few as zero species have been described in a single year, and millipede taxonomic productivity has varied drastically throughout history. The phylogenetic framework of the group at high taxonomic levels is unknown other than what can be inferred from the taxonomy and relatively few published cladistic or phylogenetic studies that lack convincing support [12]–[15]. Due to a lack of well-supported phylogenetic hypotheses, the higher-level classification that currently exists for the group is based more on pragmatism to facilitate identification rather than convey natural groupings. Consequently, the class is replete with higher-level taxa containing very few species (e.g., 68% of genera contain only one or two species!) [12]. Additionally, given the ∼12,000 described species and ∼3,000 recognized genera, genus level taxa contain four species on average [12] but range from one to over 200 (*Rhinocricus*: Spirobolida: Rhinocricidae). A paucity of interest in the recent past in millipede systematics may have reduced the numbers of taxonomic revisions being produced with a concomitant lack of progress in the development of techniques and character systems that can be employed to delimit groups above the level of species. It is surprising that an ordinal classification scheme proposed in 1895 [16] survives to this day [17] and recognizes taxa that are based on ambiguous or, in some cases, no synapomorphic characters. Only recently have phylogenetic and/or cladistic approaches been employed to evaluate the existing higher classification scheme [12]–[15], [18], [19], that is, few studies comprise sufficient taxonomic or character sampling to address a substantive restructuring of diplopod classification. Moreover, many of these studies have focused attention at the lower taxonomic levels, primarily concerned with population, species, and generic level relationships [12]–[15], [19]–[32]. Consequently, monotypic taxa based on often ambiguous or poorly defined sets of morphological characters are still erected in the absence of any formal analysis.

**Table 1:** Numbers of species, genera, and families for various arthropod groups and the average numbers of species per genus and per family.

	# of species	# of Genera	# of families	# of species per genus	# of species per family
Diplopoda	12,116	3,005	146	4.03	83
Araneae	42,055	3,821	110	11	382
Pseudoscorpions	3,433	443	25	7.7	137
Chilopoda	5,062	401	25	12.6	202

In this study we attempt to assess the uniformity of the diplopod ordinal-level taxa with regards to the underlying taxonomic diversity they contain using the taxonomic distinctness metric [2]. To our knowledge, this is the first time this method has been applied to taxa at a global scale. The overall impetus for this study is based on the general observation that the Diplopoda classification is “top-heavy” (i.e., the class contains far more order and family rank taxa than would be expected given the morphological diversity within the group) and that the class contains an unusual number of higher-level monotypic taxa. Sierwald and Bond [12] pointed out recently that 68% of millipede genera are monotypic or contain only two species. The results of these analyses are then compared to the diversity comprising the closely related orders of the Chilopoda (Arthropoda: Myriapoda) and the more distantly related order Pseudoscorpiones (Arthropoda: Arachnida). Additionally, we attempt to formulate an estimate of the global species diversity for the Diplopoda that is based on the current nominal species diversity within the group. The current estimate of millipede global species diversity, 50,000–80,000 [17], lacks empirical support (but see [33]). Statistical analyses of geographic taxonomic focus and species richness are utilized to help identify patterns of worldwide millipede diversity and research bias. Together, these methods yield surprising insights into past and present taxonomic practices within the class.

## Materials and Methods

### Diplopod Catalogue

Classification and species diversity data were extracted from a catalogue of the Diplopoda assimilated and archived at the Field Museum of Natural History (Chicago, Il) by P. Sierwald. The taxonomy of each millipede order was recorded using the traditional Linnaean classification ranks – species, genus, family, and order. Other taxonomic levels (subspecies, subgenera, tribes, subfamilies, superfamilies, and suborders) were not included in the analysis due to infrequency of use in millipedes. Species that are not currently assigned to families were included in a single placeholder taxon per order. This assignment decreases the overall average distance between species by reducing the inflation of higher taxa. Many of these unplaced species are of dubious validity and most have not been encountered since they were first described. The data set assimilated spans the time period from 1758–2007. Geographically, the data were not complete for approximately 17% (2,014 of 12,116 nominal species) of taxa.

### Taxonomic Distinctness

The taxonomic distinctness metric [2] is the average distance from any species to any other in a phylogenetic tree. The input tree can be created using phylogenetic data or, in the absence of such data, by translating the hierarchical classification scheme in place for a group. The formula is summarized as follows:
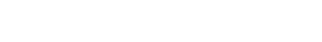
where *ω* is the “distinctness weight” (i.e., the distance in nodes when traversing the tree from one species to another) between species *i* and *j*, and *s* is the number of species in the tree. This metric was assessed for each order of millipede in the R [34] package Vegan [35] using the current millipede classification [11]. Due to the lack of a well-resolved diplopod phylogeny with complete taxonomic sampling, the input phylogeny comprised the Linnean hierarchical levels (i.,e., Class, Order, Family, Genus, Species) translated into a tree. The implementation of the taxonomic distinctness metric in Vegan scales the longest path to 0 thus all values reported herein reflect this adjustment. The taxonomic distinctness scores for all orders were plotted against their respective log transformed species totals. A linear regression was fitted to the data using the R command “lm”. Taxonomic data for the non-diplopod orders of the Chilopoda [36] and the arachnid order Pseudoscorpiones [37] were assessed using the same approach. Data points for the Pseudoscorpiones and Chilopoda were added to the plot after the regression analysis for comparative purposes.

### Global Species Diversity Estimates

Three analyses were conducted to project estimated millipede species richness. First, we employed the method described by Wilson and Costello [38] that uses a class of thinned temporal renewal models. A non-homogenous Poisson process (NHPP) is extended to the models, and estimates are made using Bayesian inference by a Markov Chain Monte Carlo (MCMC) approach. This method provides estimates of the diversity remaining to be discovered and the amount that will be discovered at any point in the future.

The second approach we employed, that of Bebber *et al.* [39], examines taxonomic productivity to assess when the rate of species descriptions will reach zero. This is assumed to be the point at which all species have been described. The number of species described at time *t* was plotted against the cumulative total of species described at time t-1. A local regression was used to assess overall trends in the taxonomic productivity using the locfit package [40] in R. An overall negative linear slope is required to achieve a total species richness estimate. The point at which the overall slope becomes negative was determined and used as the final analysis starting point. This taxonomic productivity approach to estimating global species diversity was carried out in R using a script (provided by D. Bebber). The point at which a linear regression intercepts the x-axis is considered the global species total. This method requires consistent sampling efforts through time. Deviations in taxonomic productivity can be seen as changes in the magnitude and/or sign (+/−) of the initial local regression curves slope through time. As the slope becomes negative and progresses towards an x-axis intercept, the total global diversity is assumed to be nearing complete description. However, an alternate explanation for changes in the slope of the line is inconsistent taxonomic effort.

A final method used to estimate global millipede species richness relies on an *ad hoc* extrapolation based on taxa whose species diversity is considered nearly completely described (e.g., Mammalia and Aves). The global species diversity of birds [41] and mammals [42] were taken as a ratio of the species richness in the United States and Europe because these taxa are considered nearly fully described. The European data millipedes, mammals and birds were taken from Fauna Europaea [43] to standardize the countries included in the analysis. The resulting values of global species per US species were each multiplied by the US millipede diversity to obtain estimates of global millipede richness. We used US millipede species diversity because it is the most thoroughly described fauna, perhaps equitable to the European fauna, and the data for mammals and birds are considered relatively complete and available.

### Geographic Diversity Statistics

The most recent available data for land area (excluding water bodies such as lakes, seas, etc.) and human population for all of the World's countries were downloaded from http://www.geographic.org on 23 August 2010. Only countries having at least one millipede description were included in these analyses. The “lm” command in R was used to carry out linear regression analyses on the species number data per country (i.e., the number of millipede species described from each country) as a function of: land area (km^2^), human population, and human population (excluding India and China). These analyses were carried out with all countries together and with tropical countries (at least 50% of land area within the tropics) separated from nontropical countries (less than 50% of land area within the tropics). A Welch two-sample t-test was used to compare the average numbers of species in tropical and nontropical countries, and, lastly, the numbers of species per square kilometer were calculated for tropical and nontropical countries.

## Results

### Taxonomic Distinctness

**Figure 1:**
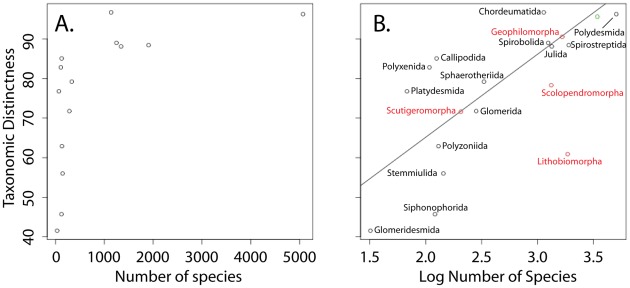
Taxonomic distinctness plots for the ordinal taxa of the Diplopoda. Plot A shows taxonomic distinctness in relation to the species diversity of the ordinal groups. Plot B shows the same relationship as the previous plot with species diversity log transformed and a best fit line obtained via linear regression (r^2^ = 0.5705, P<0.01; does not include the two millipede orders with only two species). Points representing the orders of the Chilopoda (shown in red; does not include the single species order Craterostigmomorpha) and the Pseudoscorpiones (shown in green) were added after the regression analyses and have no bearing on the best fit line.

The values for taxonomic distinctness within diplopod orders ranged from 25 (Siphoniulida – 2 species) to 96.704 (Chordeumatida – 1138 species) (Table 2). When plotted, the values appear to follow a logarithmic distribution (Figure 1A). The same taxonomic distinctness values plotted against log transformed species numbers for each order show a moderate fit to a linear regression line (r^2^ = 0.5705, P<0.01; the regression does not include the two orders with only two and three species; Figure 1B). When the chilopod orders (excluding the Craterostigmomorpha comprising a single genus) are added to the plot, all centipede points fall below or on the regression line (Figure 1B). The value for the pseudoscorpions falls near and slightly below the regression line (Figure 1B).

**Table 2:** Data used in the taxonomic distinctness regression analyses.

		*Order*	*# of species*	Δ+	*log(# of sp)*
Diplopoda-Penicillata		**Polyxenida**	108	82.81	2.03
Diplopoda-Pentazonia		**Glomeridesmida**	32	41.54	1.51
		**Glomerida**	283	71.76	2.45
		**Sphaerotheriida**	332	79.21	2.52
Diplopoda-Helminthomorpha	Colobognatha	**Platydesmida**	68	76.76	1.83
		**Polyzoniida**	130	62.9	2.11
		**Siphonophorida**	121	45.72	2.08
		**Siphonocryptida**	3	31.25	0.48
		**Siphoniulida**	2	25	0.3
	Juliformia	**Julida**	1342	88.1	3.13
		**Spirobolida**	1247	89	3.1
		**Spirostreptida**	1906	88.42	3.28
	Nematophora	**Chordeumatida**	1138	96.7	3.06
		**Callipodida**	125	85.07	2.1
		**Stemmiulida**	144	56	2.16
	Merocheta	**Polydesmida**	5070	96.26	3.71
Chilopoda	Notostigmomorpha	**Scutigeromorpha**	206	71.61	2.31
	Pleurostigmomorpha	**Lithobiomorpha**	1861	60.89	3.27
		**Craterostigmomorpha**	1	0	0
		**Scolopendromorpha**	1328	78.3	3.12
		**Geophilomorpha**	1665	90.53	3.22
Arachnida		**Pseudoscorpiones**	3432	95.62	3.54

The diplopod orders Siphonocryptida and Siphoniulida and the chilopod order Craterostigmomorpha were excluded from the plots and regressions. Δ+ = taxonomic distinctness as defined by Clarke and Warwick [2].

### Global Species Diversity Estimates

**Figure 2:**
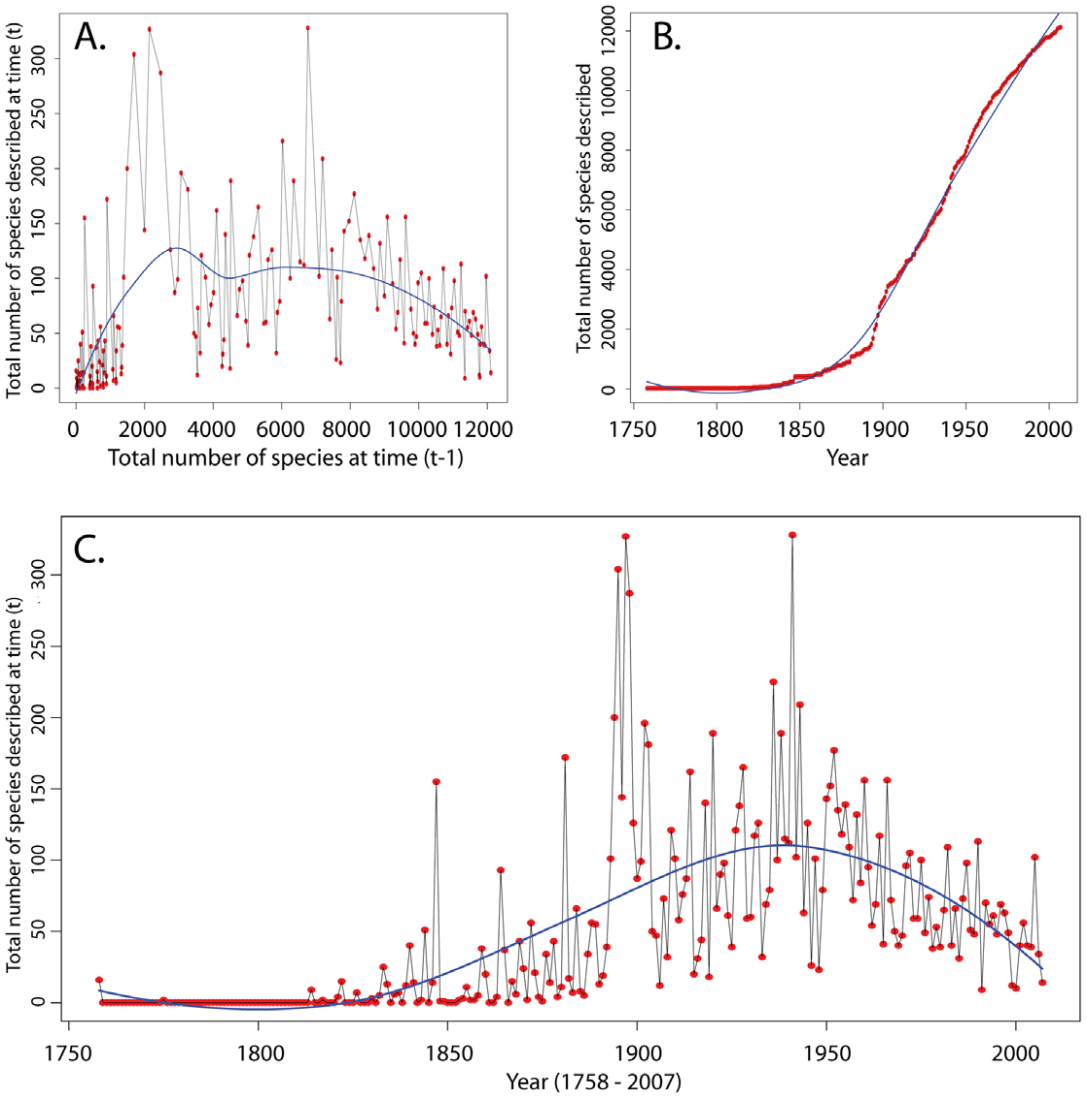
Plots showing millipede taxonomic effort over time in terms of species descriptions. Plot A shows the data as used to calculate global species diversity following the methods of Bebber *et al.* [39]. The y-axis corresponds to the number of species descriptions in a given year (t) while the x-axis shows the number of species accumulated at time “t-1”. A local regression line is shown fitted to the data. Plot B shows a species accumulation curve fitted with a local regression line with no trend toward asymptote. Plot C shows the taxonomic productivity over time in terms of species descriptions published yearly and is fitted with a local regression line.

The Bayesian methods of Wilson & Costello [38] were unable to provide an estimate of the total global diversity of millipedes due to a lack of an asymptote in the rarefaction curve (Figure 2B). Instead, the methods were used to make estimates of the numbers of species to be described between 2009 and two points in the future – 2050 and 2100. Associated 95% confidence intervals were assessed from the posterior distribution of estimates. The resulting estimates were 2030 (1820–2260) for 2050 and 4460 (4110–4830) for 2100 (Table 3). The methods of Bebber *et al.* [39] rely on taxonomic productivity to make estimates concerning global species diversity. The taxonomic effort through time appears quite uneven, dominated by early high productivity and more recently a lack of productivity (Figure 2A). Consequently, the resulting estimate from the year 1900 onward, with 95% confidence intervals, is 14495.65 (13412.94–16760.49). The ratio of bird global diversity to US diversity was 11.07:1 for the USA and 11.98:1 for Europe. Mammals had a ratio of 12.47:1 for the USA and 13.41:1 for Europe. Using these ratios, global millipede richness is estimated to be 13,671.45–20,519.18 species.

**Table 3:** Results from the millipede global diversity estimation analyses. Values in parentheses are 95% confidence intervals.

*Method*	*Point Estimate*	*2050 Additional Species Estimate*	*2100 Additional Species Estimate*
**Wilson & Costello - Bayesian**	N/A	2,030 (1,820–2,260)	4,460 (4,110–4,830)
**Bebber et al. - Productivity**	14,495.65 (13,412.94–16,760.49)	N/A	N/A
**Mammal (12.47:1) Extrapolation**	15,400.45 – NA 20,519.18 – Europe	N/A	N/A
**Avian (11.07:1) Extrapolation**	13,671.45 – NA 18,317.42 – Europe	N/A	N/A

### Geographic Diversity Statistics

All regression analyses were significant (p<0.05) but had low to intermediate r^2^ values (0.06421–0.5860). In all cases, the nontropical dataset has the lowest r^2^ values. Weaker nontropical relationships are most likely due to the need to further partition the dataset to account for latitudinal variations in ecological and climatic factors.

#### Land Area Regressions

**Figure 3:**
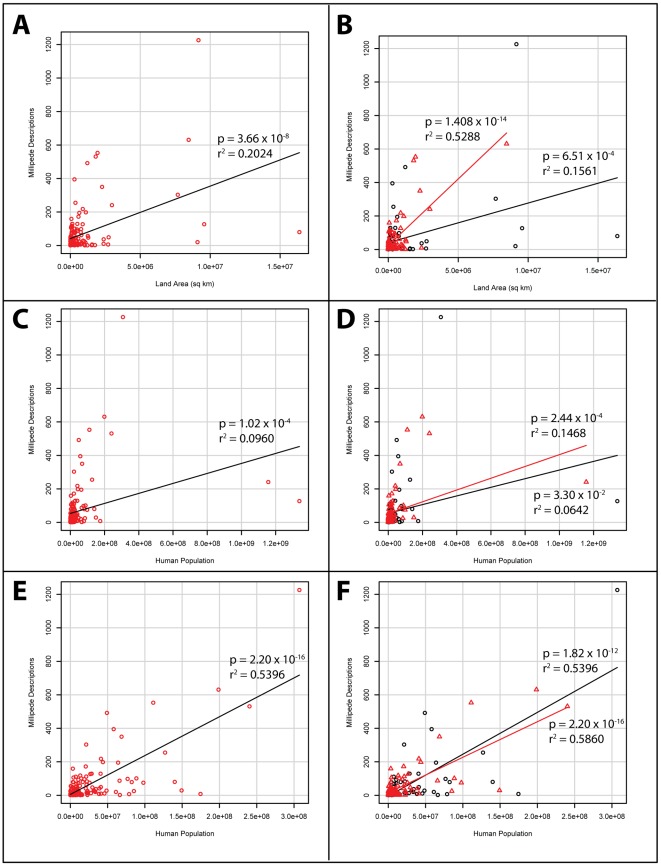
Number of millipede species descriptions in countries as a correlate of land area and human population. Plots A, C, and E show all countries from which millipedes have been described. Plots B, D, and F have the countries designated as tropical (≥50% of total land area in the tropics) or nontropical (<50% of total land area in the tropics) and were analyzed separately. The outliers for human population, India and China, are excluded from plots E and F.

The regression of a country's number of species by its land area in the combined dataset (tropical & nontropical countries included) showed a weak relationship (r^2^ = 0.2024) (Figure 3A). When separated into tropical and nontropical datasets, the result in tropical countries becomes much stronger (r^2^ = 0.5288) while that of nontropical countries becomes weaker (r^2^ = 0.1561) (Figure 3B). It is likely that latitudinal species diversity variation within the nontropical countries weakens the overall results. Further splitting of the nontropical dataset could counteract this effect.

#### Human Population Regressions

The regression of a country's number of species by its human population revealed weak relationships for the combined (r^2^ = 0.09602), tropical (r^2^ = 0.1468), and nontropical (r^2^ = 0.06421) datasets (Figure C–D). However, when India and China are removed from the datasets, due to their tremendous human populations, the results strengthen (combined r^2^ = 0.5396, tropical r^2^ = 0.5860, and nontropical r^2^ = 0.5208) (Figure 3E, F).

The average numbers of species described from tropical (65.58) and nontropical (66.80) were not significantly different (t = 0.0523, df = 124; p = 0.9584) (Table 4). The number of species descriptions per square kilometer in tropical (1.246×10^−4^) and nontropical (6.083×10^−5^) differed by a factor of 2.4 (Table 4).

**Table 4:** Results from the estimation of millipede species diversity in tropical and nontropical countries.

	*Tropical*	*Nontropical*
**# of Species**	4743	5311
**Average # of Species per Country**	65.57	66.8
**Global Land Area (km2)**	77971681.7	42630129.9
**Species per km2**	6.0830×10^−5^	1.2458×10^−4^

Countries were defined as tropical if half the total land area of the country is within the tropical latitudes.

## Discussion

As discussed in the introduction, a classification system wherein taxonomic ranks represent similar amounts of diversity is an asset to many various types of biological investigations (e.g., community ecology, studies of rates of evolutionary diversification, etc.). Studies comparing hierarchical ranks that lack equivalency are problematic by definition. If higher taxonomic categories in which species are placed are to be more than just simply placeholders, they must conform to some common metric by which they are delineated. In the study presented herein, we have shown that the ordinal-level taxa contained within the class Diplopoda are not equivalent based on a metric of taxonomic distinctness that is calculated from the classification-based tree. Such a disparity raises the question of whether the higher taxonomic ranks within and between millipede orders, and the orders themselves, are phylogenetically equivalent. Unfortunately, we cannot address the question of phylogenetic equivalence until a robust phylogeny is available for all millipede higher taxa, but these data suggest that a phylogenetic-based classification scheme will likely differ sharply from the existing. In addition, it is our contention that the millipede orders are overinflated with taxa above the species level when compared to other arthropod groups; that is, the orders contain far more families and genera than predicted. These disparities may further be exacerbated by the unequal treatment of worldwide millipede research (focused largely on North America and Europe) demonstrated in our attempts to estimate global species diversity. Many geographic areas lack sufficient sampling such that reliable estimates cannot be made. For example, the tropics are vastly understudied in relation to the diversity that is estimated to be present. Millipedes are an example of a mega-diverse yet understudied taxon that exhibits attributes (inconsistent taxonomy, geographic research biases, etc.) that are likely common in such understudied taxa. Because of recent efforts to assemble a catalog for the millipedes, we are now able to identify these potential shortcomings and areas in need of future work.

While a number of potential causes of taxonomic inequality exist, we have attempted to explore many of these causes with the available data. We list here the potential causes of the taxonomic “problems” we deemed most likely applicable to the Diplopoda:

- **The group is poorly studied and character systems are not well developed.**
  Given that apomorphic characters define fewer than 50% of higher taxa, this appears to be a major issue [12].
- **The group is very old, and ancient extinctions have occurred**
  Fossil data has confirmed the ancient age of the Diplopoda [44]. Couple this known antiquity with the disjunct distributions of some extant taxa (e.g., the North American – Asian disjunct platydesmidan genera *Brachycybe* and *Okeanobates*; the genus *Hirudicryptus* found in the Canary Islands, Madeira, Nepal, and Taiwan) [45], and we have circumstantial evidence to support this as a potential cause of some millipede taxonomic issues. Furthermore, all Paleozoic fossils are not attributable to extant orders, indicating that these fossils represent high-level taxa that went extinct long ago. However, until proper molecular divergence time estimation analyses can be undertaken for the breadth of millipede diversity, we cannot support or reject hypotheses concerning divergence times of extant orders, families, or genera. Even then, detecting extinctions using molecular phylogenies is an unresolved issue [46].
- **Taxonomic and sampling efforts have been inconsistent with regards to taxa and geography**
  Analyses of geographic diversity reported herein demonstrate that the European and North American fauna have enjoyed a disproportionate amount of attention. Additionally, the taxa comprising the subterclass Eugnatha, those species with diagnostic gonopods, are much more thoroughly studied than taxa lacking these species-specific sexual features.
- **Primary homology hypotheses based on poorly defined character ontologies are incorrect or underdeveloped**
  This is known to be true concerning millipedes [12]. A prime example concerns the male sperm transfer appendages, the gonopods. When characteristics of the gonopods are used to reconstruct higher-level relationships, assumptions concerning the homology of often highly derived morphologies have never been addressed empirically.
- **Questionable nomenclatural practices.**
  Through taxonomic history, many taxa have been left orphaned; that is, genera are not assigned to families while, in some cases, the placement of genera in tribes and subfamilies lack substantiation by characters. Furthermore, even recently published studies use conflicting taxonomies and alternate taxonomic hypotheses are ignored and often not even cited. For example, the validity of the paradoxosomatid genus *Asiomorpha* Verhoeff, 1939 is of questionable validity as its type species, the widely distributed and highly invasive species *A. corarctatus* De Saussure, 1860, has been synonymized under *Orthomorpha* Cook, 1911 repeatedly, only to be treated as valid shortly thereafter. However, the genus *Asiomorpha* is still treated as valid or as a synonym by other researchers just during the past five years [Sierwald, in prep].
- **Violation/abandonment/rejection of accepted analytical concepts (e.g., molecular phylogenetics and revisionary taxonomy)**
  Only recently have published studies employing molecular phylogenetics to reconstruct relationships within the Diplopoda been undertaken. Additionally, our literature analysis demonstrates the lack of revisionary thinking and context when erecting novel taxa (see below).

Although not all potential causes of taxonomic issues have been examined directly, past taxonomic practices appear to have worked in concert with the characteristics of millipede history (e.g., ancient origins, vast diversity, morphological and ecological stasis in many groups, etc.) to yield a classification replete with problems. Some of these issues may never be fully accounted for, but taxonomic practices can be better partitioned amongst taxa and geographic regions and use a more complete suite of tools. By doing so, we can minimize the inconsistencies and difficulties of working with millipede taxa. We explore a number of these issues in detail below.

### Utility of catalog data

The use of catalog data is becoming increasingly more common when investigating large-scale patterns of biodiversity. Resources like the Global Biodiversity Information Facility (GBIF) make these databases accessible and greatly facilitate their usefulness. When investigating organisms with low vagility and restricted ranges, like millipedes, the resolution of these data can be increased to a point at which specific questions regarding the diversity and distributions of taxa can potentially be addressed. Biological catalogs contain vast amounts of information concerning taxonomic effort, biodiversity hotspots, and species habitat preferences, and are thus valuable tools for studying evolutionary diversification. This work demonstrates the efficacy of one such catalog of information - the World Millipede Catalog [Sierwald, in prep]. Using only these data coupled with readily available data on the Earth's political divisions and human populations, we have analyzed the uniformity of the ordinal taxa, attempted to estimate the global species richness, and conducted statistically supported investigations of the patterns of diplopod species diversity as we understand it. These methods have yielded interesting results concerning the global patterns of currently recognized millipede diversity and potentially alarming conclusions about the state of millipede taxonomic expertise and research.

### Taxonomic Distinctiveness

As discussed in the introduction, many biological investigations rely on a well-supported classification that reflects phylogeny. The results of this study indicate that the higher taxonomic classification scheme for millipedes is uneven and potentially inflated (i.e., species are more separated taxonomically than would be expected). Taxonomic research that historically focused on the species level resulted in classification scheme that disregards phylogeny in lieu of simply facilitating identification and diagnosis. On 15 September 2010 an ISI database literature search for the topics of “Diplopoda” AND “taxonomy” spanning the past decade (2000–2010) was conducted, and the number of family or genus level revisions is far outweighed by piecemeal descriptions. Of the 155 works that were considered taxonomic in nature (a number of which are reviews of regional fauna making this a conservative estimate), 81 erected new taxa (many of which were single taxon descriptions) and were not revisionary works. Because revisions that would potentially synonymize taxa are lacking, an emphasis on “narrow scope” taxonomy may be driving the proliferation of higher taxa.

Millipede orders do not reflect similar amounts of diversity when measured as the average taxonomic distance between species within an order ( = taxonomic distinctness). The groups' taxonomic distinctiveness values, when plotted against the log of the number of species in the respective orders, show only a moderate relationship (r^2^ = 0.5705; Figure 1B). The disparity between orders may be due to the over-inflation of higher taxa in some highly studied groups (e.g., the orders Chordeumatida and Callipodida) and the existence of “dumpster” taxa in poorly studied groups (e.g., the family Julidae and the orders Siphonophorida, Glomerida, and Polyzoniida). Over-inflation increases the distance between species and an order's taxonomic distinctness value while “dumpster” taxa deflate distinctness. Both ends of the spectrum are present within and between orders resulting in an ordinal level classification comprising unequally treated and inconsistent units. The remaining variation, approximately 43% of the total, could be caused by a number of factors that are discussed below.

The taxa that deviate most from the regression line are those groups that appear to be the most neglected. The orders of the Colobognatha, Pentazonia, and Penicillata (the former lacks species-level diagnostic characters of their sperm transferring appendages and the latter two do not have these structures at all) are least diverse in terms of species richness. Additionally, other groups are ignored because of geography (e.g., the order Stemmiulida). As a result, we postulate that the lack of revisionary work in these groups, due to the difficulty of working with them compared to others, may have produced these results. A lack of synthetic taxonomic revisions employing new methodological approaches coupled with a paucity of taxonomic resources has led to groups that are practically ignored by today's research community.

As mentioned above, classifications are influenced by a group's history and are therefore not likely directly comparable between taxa. Herein, we compared the taxonomic distinctness of related arthropod taxa to the millipedes to ascertain the relative differences between these groups. Despite the intrinsic differences between these taxa, we demonstrate an overall trend for the millipedes to be “over-inflated” with higher-taxa. When the four relevant orders of the Chilopoda and the arachnid order Pseudoscorpiones were added to the millipede regression plot (Figure 1B), all non-diplopod orders fell on or below the regression line. It is important to note that the regression analysis was not rerun – the points for the additional orders were simply overlaid onto the existing plot. One interpretation of these results is that the millipede orders are indeed over-inflated (e.g., too many higher taxa in relation to species diversity) as a whole compared to both related groups (Chilopoda) and an unrelated arthropod order (Pseudoscorpiones) (Table 1). As mentioned above, this is expected in a classification that is focused on the erection of new species and higher taxonomic groups in the absence of large-scale revisions. However, some millipede groups show evidence of cryptic diversity [21] (but see [47]) thus species constructs based only on traditional morphological character systems may alternatively result in underestimations of species numbers. By adding more species to existing higher taxa (i.e., cryptic species), the average taxonomic distance between species might decrease, and the regression line would move down the y-axis, as the ordinal taxonomic distinctness values decreased, and approach those values of the non-millipede taxa that were included in this analysis.

Alternatively, the apparent over-inflation of taxa within the Diplopoda could be due to underestimated species numbers in the non-diplopod taxa included in the analysis. Much of myriapod (i.e., Diplopoda) and arachnid (i.e., Araneae and Opiliones) taxonomy is based on diagnostic sperm transferring appendages that differ in structure as a result of reinforcement of mating isolation via sexual selection by female choice [48], [49] or antagonistic coevolution (see [50]). Because centipedes and pseudoscorpions do not share similar genitalia-based character systems, much unrecognized cryptic species diversity might likewise exist within these groups. However, not all millipede groups have informative gonopod structures; only the orders comprising the Eugnatha (Polydesmida, Stemmiulida, Chordeumatida, Callipodida, Siphoniulida, Spirosteptida, Spirobolida, and Julida) have sperm transferring gonopods that are heavily modified and diagnostic at the species level. The orders of the Colobognatha (Platydesmida, Polyzoniida, Siphonophorida, and Siphonocryptida) have sperm transferring gonopods that are less modified, leg-like, and non-diagnostic while the Pentazonia (Glomeridesmida, Glomerida, and Sphaerotheriida) and the Polyxenida lack modified anterior copulatory legs altogether. Three of the seven included millipede orders that do not have diagnostic gonopods are points above the regression line (Polyxenida, Sphaerotheriida, and Platydesmida; Figure 1B). Despite the lack of sexual characters on which to base species level identifications, these millipede orders still appear overinflated at higher taxonomic levels with respect to the included non-millipede taxa that also lack diagnostic sexual structures.

A final explanation for the apparent inequality between the orders is that the current taxonomy does, in fact, reflect the diversity that exists within the Diplopoda. Given the age of the orders (as evident in the fossil record; reviewed in [44]), extinction within and between extant millipede lineages could produce a pattern of variable taxonomic distances between species. The current oldest known land animal was a millipede-like animal dating to 428 MYA [51], and fossils of the highly derived millipede superorder, Juliformia, are present from as early as the Pragian Age of the Lower Devonian (∼410 MYA) [52]. Very old lineages may have a few relict taxa that comprise monotypic families or genera. As a result, the taxonomic distinctness values for the taxa containing these groups would only appear inflated. Most ancient millipede fossils are not representative of extant orders and, as a result, only provide information on the timing of cladogenesis of extant superordinal taxa and higher [44]. As a result, these fossil data cannot be used to estimate the divergence times of lower taxa without a robust molecular phylogeny in place.

A recent work by Shelley and Golovatch [45] posits a number of hypotheses regarding the timing of divergence and biogeography of the major diplopod groups. However, the work lacks any statistical or phylogenetic analyses. Means to objectively assess the issues of millipede divergence dating (e.g., molecular divergence dating software like BEAST [53], r8s [54], and PhyloBayes [55]) and historical biogeography (e.g., Lagrange [56], PhyloMapper [57], and RASP [58]) are available and should be employed in subsequent investigations.

Assuming the millipedes are “over-split”, systematists must work toward a classification that reflects phylogeny and consists of equally treated and uniform units. Retroactively, researchers can revise existing groups with more of an emphasis on phylogeny. As mentioned above, comprehensive revisionary work in millipedes has recently been lacking when compared to the erection of single or a few taxa (this is not to say that revisionary work is not being done, just at a much lower rate than studies of a more limited scope). While the description of a single species does provide valuable information, higher taxonomy suffers when the documented diversity of related taxa is not taken into account. In addition, diplopodologists would be well served if more revisionary works were published, as many taxa, of all ranks, have not been investigated formally since their original descriptions. Modern phylogenetic methods help to reveal cryptic diversity while providing an evaluation of the validity of higher taxa. This is not to say that alpha taxonomy should be undervalued but that caution with regards to erecting higher taxa should be exercised when species are *not* considered in some broader context.

### Global Species Diversity Estimates

We clearly lack a robust estimate of just how many millipede species exist. Our species estimates were surprising given the often-quoted number of 80,000 [17]. Two of the methods (taxonomic productivity-based method of Bebber *et al.* and extrapolation from other taxa) employed here achieved similar numbers of ∼13,000–15,000 global millipede species, but the third method (Bayesian method of Wilson *et al.*) was unable to derive an estimate. The inability of the Bayesian method to infer the total global diversity of millipede species is due to the lack of an asymptote in the rate of species description accumulation. Contrasting this with the results of the analysis relying on the methods of Bebber *et al.* (14,495.65 total species) and the estimates obtained by extrapolation from nearly complete faunas (13,671.45–18,317.42 from birds; 15,400.45–20,519.18 from mammals) yields interesting results. The latter two methods seem to indicate that, given the >12,000 nominal millipede species, the described diversity of the Diplopoda is nearing completion. However, because various regions of the world (e.g., New and Old World Tropics) are vastly understudied and no asymptote is seen in the rate of species description accumulation (Figure 2B), this is not a likely scenario. Another explanation for this result is inconsistent taxonomic effort. As evident in Figure 2A, taxonomic productivity within the Diplopoda has been quite variable over time. In the past, researchers described more than 300 species in a single year. Towards the present, fewer species have been described per year due to a paucity of researchers working in the field ( = less taxonomic effort) and a shift toward more modern taxonomic practices (i.e., molecular phylogenetics). Because the methods of Bebber *et al.* rely on consistent overall effort, these results are likewise suspect.

Another conclusion that could be drawn from the taxonomic productivity-based diversity estimation is that, instead of the total number of millipede species worldwide, the x-intercept represents the point at which the field of millipede taxonomy will effectively end. Given the current trends of fewer species being described each year (Figure 2A) and the projections of this method, the extinction of diplopod taxonomic expertise could be at hand. Recent attempts to provide training to a new generation of diplopodologists have benefitted from the United States National Science Foundation PEET program as this initiative has trained a number of taxonomists in recent years (far more than in the past). However, whether these and other initiatives will be sufficient to increase interest in the group remains to be seen. We have seen an increase in international millipede focus as well. Newly trained diplopodologists from Africa, Europe, and Asia have made valuable contributions in recent years, but the general trend of too few experts continues.

A final interpretation of the species diversity estimates reported herein is that biases in the methods used to discover millipede species and the regions in which efforts have been focused have led to a paradigm that impedes progress toward understanding global diplopod diversity. The reliance on morphology while largely neglecting the modern tools and approaches (e.g., molecular phylogenetics) could be causing the apparent plateau in millipede descriptions in well-studied areas (i.e., the United States and Europe). Evidence for this scenario is compelling in taxa with large ranges, extremely low vagility, and nondiagnostic genitalic morphology. Orders that comprise the Colobognatha, Pentazonia, and Penicillata can be described as such and have been neglected by systematists because of the paucity of diagnostic morphological characters. As a result, these groups are quite divergent in terms of the taxonomic distinctness metric (Figure 1B; Table 2). Additionally, there are taxa within the orders that usually have diagnostic gonopods that cannot be diagnosed using their genitalia, such as members of the genera *Pachyiulus* (Julida: Julidae) [59] and *Anadenobolus* (Spirobolida: Rhinocricidae) [20]. We must employ modern, molecular techniques to further our knowledge of such groups.

Besides ignoring readily available sources of diagnostic characters, millipede systematists have neglected many parts of the World, namely the tropics. In both the New and Old Worlds, millipede taxonomic endeavors have focused on temperate, northern hemisphere taxa. The diversity of Europe and North America is better understood than anywhere else in the world. This continues today despite the acknowledgement among experts that a rich tropical millipede fauna remains woefully understudied [12]. The temperate areas of the southern hemisphere, such as much of Australia, have received little attention in recent years. If millipede taxonomic expertise is in danger of extinction, as Figure 2A may indicate, we must move past the conventions of the past and embrace new technologies. Understudied geographic areas provide exciting new frontiers for diplopodology that could be alluring to a new generation of experts. In the end, by shifting our paradigm, we could both achieve a better understanding of the World's millipedes and rescue our field from expiration.

### Geographic Diversity Statistics

The relationship between geographic species diversity data and human population and land area data provide both expected and difficult to interpret results. Global regressions involving land area were all understandably weak given climatic heterogeneity between tropical and nontropical countries. Weak results involving nontropical countries could be the result of increased climatic variability (i.e., higher latitudes are much colder and therefore less productive). While the tropical countries have more consistent climates, nontropical countries vary more and are certainly not equal units. Merely a moderate relationship between land area and species diversity in tropical countries could be interpreted as a lack of effort in these regions historically. When the population outliers of India and China are removed, the results between human population and species descriptions strengthen considerably. This result is reasonable given human population centers and the focus of millipede taxonomy on northern temperate regions.

The apparent equality between millipede species description accumulation in the tropics versus nontropical countries is perplexing. There is more land area in nontropical countries, but they are much less productive (especially nearing the poles). When the numbers of species descriptions per square kilometer in the tropics and nontropical regions are compared, there is an order of magnitude more in tropical countries. Given the lack of focus on tropical taxa, we expect this discrepancy to grow. Evidence of this can be seen in Figure 3B where millipede descriptions per square kilometer increase at a much faster rate in the tropics than in nontropical countries. This is further evidence that the tropics need more attention from diplopodologists.

### Conclusions

In a recent work [60], the efforts of the global millipede community are championed, and it is stated that millipedes will soon be able to discard the monicker of “poorly known”. While it is true that some strides are being made regarding millipede systematics, practices of the past have left us with a classification that suffers from many problems. A legacy of “piecemeal” taxonomy that employs a morphological divergence argument to validate the erection of new higher taxa and the disregard for taxa or geographic regions (mostly due to limited resources and hostile political situations) have all contributed to the results discussed above and continue to hinder the efforts of junior colleagues. Concomitantly, our lack of understanding concerning the ages of groups and the relationships between them render us unable to discriminate the causes (though a cumulative effect of all possibilities seems likely). Finally, the lack of availability of millipede literature, as a result of language barriers or obscure/extinct journals, makes the learning curve associated with many millipede groups quite high. While students have a great deal of opportunity to revise poorly treated groups, they are slowed by the lack of resources (i.e., good descriptions, taxonomic keys, and a complete catalog to species, etc.). Instead of working on new frontiers of millipede taxonomy, younger researchers are spending time sorting out groups that have been studied previously.

The taxonomic distinctness metric, usually used in community ecology, is applied here to measure similarity between taxa. In terms of higher taxon inflation, we show that the diplopod orders do not share similar levels of diversity at the ordinal level even when discrepancies in species numbers are accounted for. In addition, we demonstrated that the millipede orders, as a whole, appear more overinflated than other arthropod groups. Whether the ordinal taxa of the Diplopoda are inflated due to poor taxonomic practices or accurately reflect the diversity contained within the class (given the extreme age of higher taxa) is difficult to say. Admittedly, data on more non-diplopod orders would have helped to strengthen any conclusions, and further investigations could elucidate interesting trends concerning millipede diversity.

Our attempts to achieve a global estimate of millipede species diversity yielded interesting results. Because the assumptions of the taxonomic productivity analysis were violated and no asymptote was reached in the Bayesian analysis, we cannot make a confident estimate. Estimates of total species diversity can be informative even if a plausible estimate cannot be obtained. We demonstrated here that two recent, statistically based methods of global diversity estimation produced very different results while a traditional extrapolation approach yielded results that were consistent with one of the previously mentioned methods. When these results were further analyzed, a potential issue with the way in which diversity is described within a group came to light. Our interpretation is that a paradigm shift in millipede systematic practices is necessary to ensure the survival of our field and to accurately assess diplopod biodiversity. Millipede systematists need to embrace new character systems and methods while expanding the geographic scope of their work. Biases toward character systems and focal taxa are evident in the taxonomic distinctness results while a need for further focus on differing geographic regions, especially the tropics, is evident in the geographic analyses.

All said, it is our opinion that the often-quoted estimate of 80,000 species lacks support. While not implicitly stated in the manuscript, the recent estimates of Mora *et al.*, [33] show a range for the Diplopoda that appears to include 80,000 species. However, the millipedes are considered a taxon with “near complete inventories”. We have demonstrated here that this is not the case, and the estimates of Mora *et al.* suffer their own deficiencies. It is disingenuous of millipede workers to continue to state this number as fact or as an estimate that carries the gravitas of actual data. Based on our assessment of the data and the current state of millipede taxonomy, we would suggest that this number be scaled back to a more moderate estimate of 15,000–20,000, thus accounting for the understudied nature of many taxa and geographic areas. This number may indeed be an underestimation of global millipede diversity stemming from incomplete data, and it should be taken as such.

Because ecological, evolutionary, and other studies rely on consistent taxonomy, it is clear from this study that the equality of higher taxa should not be assumed for any group. As demonstrated here, considerable variation can exist, even at levels as distinguishable as orders. This variation may or may not be due to natural variation. Taxonomic practices within a group and the neglect of phylogeny in exchange for a classification that facilitates identification may lead to issues of inconsistency between taxa. Contrary to the conclusions drawn by Shelley [57], millipede taxonomy is still, methodologically speaking, in its infancy and has not benefitted from modern phylogenetic thinking and approaches to delineating higher taxa. Indeed, it remains unclear to us as to what constitutes a higher taxonomic group within the Diplopoda and what information is used in making these delineations; in the absence of phylogeny, there appears to be no consistency in what data are used to establish groups above the species level. To date, a phylogeny that includes great enough taxon sampling and/or characters to draw well-supported conclusions is lacking. The availability of molecular data can help to rectify the difficulties associated with using millipede morphology at high-levels (e.g., homology). Because, like millipedes, many other mega-diverse groups may suffer similar neglect, researchers should be aware of these issues when planning experiments using such taxa.
